# Potential influence of anaesthesia techniques on the recurrence and progression after resection of non-muscle-invasive bladder cancer: a propensity score-matched analysis

**DOI:** 10.1186/s12871-022-01802-6

**Published:** 2022-08-18

**Authors:** Ruifeng Xue, Chongxi Zhao, Dongtai Chen, Peizong Wang, Wei Xing, Weian Zeng, Qiang Li

**Affiliations:** 1 Department of Anaesthesiology, State Key Laboratory of Oncology in South China, Collaborative Innovation Center for Cancer Medicine, Sun Yat-sen University Cancer Center, Guangzhou, 510060 Guangdong China; 2 Department of Anaesthesiology, State Key Laboratory of Ophthalmology, Zhongshan Ophthalmic Center, Sun Yat-sen University, Guangzhou, 510060 Guangdong China

**Keywords:** General anaesthesia, Epidural anaesthesia, Primary non-muscle-invasive bladder cancer, Recurrence-free survival time, Progression

## Abstract

**Background:**

The non-muscle-invasive bladder cancer is a common malignancy of the urinary system. Many patients relapse after transurethral resection surgery. Different anaesthesia techniques may influence a patient’s immune system during the perioperative time. In this study, we examined the effects of different anaesthesia techniques on the prognosis of primary non-muscle-invasive bladder cancer after transurethral resection surgery.

**Methods:**

In the period 2008 to 2017, a total of 926 patients suffered primary non-muscle-invasive bladder and underwent transurethral resection of bladder tumour surgery for the first time. These patients were divided into two groups according to the techniques that were used. There were 662 patients in the general anaesthesia group, who received propofol, opioid drugs (fentanyl family), non-depolarizing muscle relaxants, and sevoflurane, and 264 patients in the epidural anaesthesia group, who had an epidural catheter placed in the L2-L3 or L3-L4 interspace with a combination of lidocaine and ropivacaine or bupivacaine. We analyzed the influence factors that might affect prognosis and compared the recurrence-free survival time and the progression between the two groups.

**Results:**

The differences between the two groups in recurrence rate and progression rate were not statistically significant. Progression-free survival time of the epidural anaesthesia group was longer. Multivariate regression analysis showed that anaesthesia techniques were not independent influencing factors for recurrence and progression.

**Conclusions:**

It was not found that anaesthesia techniques affected the recurrence or progression of patients with primary non-muscle-invasive bladder cancer after transurethral resection of bladder tumour.

## Background

Urinary bladder cancer can be classified into non-muscle-invasive bladder cancer and muscle-invasive bladder cancer. The 5-year survival rate of urinary bladder cancer varies between 97% (stage T1) and 22% (stage T4) [1]. Cigarette smoking, occupational carcinogen exposure [2], analgesics [3], thiazolidinediones [4], and genetic factors are associated with risk of urinary bladder cancer [5]. According to the WHO 2017 TNM classification of urinary bladder cancer, non-muscle-invasive bladder cancer includes both mucosa (stage Ta or CIS) and submucosa (stage T1). The non-invasive papillary carcinoma stage is Ta, carcinoma in situ is CIS, and stage T1 indicates that the tumor has invaded the subepithelial connective tissue [6]. TURBT is a mainstay treatment for bladder cancer, especially non-muscle-invasive bladder cancer. Depending on the histological grade and TNM stage of the tumor, the surgeon will determine whether further adjuvant therapy, including intravesical chemotherapy and BCG immunotherapy, is needed following TURBT surgery [6]. Among patients with primary non-muscle-invasive bladder cancer, approximately 30% suffer recurrence after the first time of TURBT.

Both the dissemination of tumour cells and suppression of immune system function can result in recurrence of cancer [7]. The results of several retrospective studies have indicated that different anaesthesia techniques can influence the recurrence-free survival time or overall survival time [8–11], as anaesthesia techniques may affect the immune system and the surgery-related stress response. A systematic review and meta-analysis published in 2015 suggested that regional anaesthesia and analgesia might improve overall survival but not reduce recurrence after oncologic surgery [12]. In 2019, a randomized controlled trial about the recurrence of breast cancer after regional or general anaesthesia published in the Lancet showed that regional anaesthesia-analgesia didn’t reduce the recurrence rate of patients who underwent potentially curative surgery, although regional anaesthesia-analgesia alleviated the perioperative factors such as the surgical stress response, use of volatile, and opioid for analgesia, which could weaken host’s defence against recurrence [13].

A retrospective study of non-muscle-invasive bladder cancer in 2017 shown that spinal anaesthesia was associated with a lower recurrence rate and a longer recurrence-free survival time. However, only 231 patients were included in this study, and many of them accepted more than one time of transurethral bladder tumor resection [14]. The results of this study might be distorted by a number of confounding factors. Therefore, it is worthwhile to investigate whether anaesthesia techniques can influence recurrence among more patients undergoing TURBT for the first time.

## Methods

### Patients

We retrospectively analysed the patients who underwent TURBT at Sun Yat-Sen university cancer centre (Guangzhou, PR China) from January 2008 to May 2017. The endpoint of this retrospective study was June 2020 or the first time of recurrence, so that patients could be followed up for at least 3 years.

### Data collection

Basic patient information included age, gender, height, weight, hypertension and diabetes mellitus. Tumour condition and treatment measures included TNM stage, grade, risk group, size of tumour, time of surgery, anaesthesia techniques and drug, status of intravesical therapy, number of tumour. The follow-up visits were time of recurrence and status of progression.

#### Inclusion and exclusion criteria

The inclusion criteria were primary non-muscle-invasive bladder cancer undergoing TURBT for the first time. The exclusion criteria included patients under 18 years old, patients with CIS, recurrent or metastatic bladder cancer, tumour had invaded the muscle layer of bladder, those with other kinds of cancer, and those who died of diseases other than bladder cancer.

### Recurrence and progression

Recurrence was defined as the first disease relapse in the bladder proved by histopathologic diagnosis regardless of tumour stage. Progression was defined as tumour recurrence at higher tumour stage, or an increase in grade.

### Anaesthesia techniques

Induction of general anaesthesia was completed by three kinds of drugs: propofol (2–3 mg/kg), fentanyl (3–4 μg/kg), cis-atracurium (0.15–0.2 mg/kg), remifentanil and sevoflurane were used during the surgery. Patients received epidural anaesthesia with a combination of lidocaine(2%) and ropivacaine(0.5%) or bupivacaine(0.375%). The epidural catheter was placed in the L2-L3 or L3-L4 interspace. Depending on whether the patient has a contraindication to epidural or general anaesthesia, the type of anaesthesia is determined by the primary anaesthesiologist. In actual clinical practice, the choice of anaesthesia techniques also taken into account the surgical needs.

### Statistical analyses

Statistical analyses were performed using SPSS 19.0 (SPSS, Chicago, IL). Continuous variables were analysed by Student’s *t*-test, and the χ2 test was used for categorical variables. Survival analyses (RFS and PFS) was performed by Kaplan-Meier survival estimates, and compared by log-rank test. Cox regression analysis was used to assess factors related to the outcomes of interest. The factors with a *P* < 0.05 in the univariable Cox regression analysis and some factors associated with the prognosis of disease were entered into a forward multivariable Cox regression model to test for independent association. Propensity score matching analysis was used to allow an unbiased comparison, based on all variables including age, height, weight, gender, hypertension, diabetes mellitus, TNM stage, grade, tumour size, number of tumour, risk group, intravesical chemotherapy. The caliper was 0.1. Patients were matched at a ratio of 1:1. *P* ≤ 0.05 was taken to be statistically significant.

### Ethics

The Institutional Review Board of Sun Yat-Sen University Cancer Center approved this retrospective study of clinical data, which was conducted in accordance with the principles of the Declaration of Helsinki. The need for informed consent was waived due to the retrospective nature of the study.

## Results

### Baseline information before and after the propensity score matching

From January 2008 to May 2017, 1125 patients with primary non-muscle-invasive bladder underwent TURBT surgery. A total of 986 patients matched the inclusion criteria, and they were divided into two groups based on the anaesthesia techniques applied. Twenty-four patients were lost to follow-up results, and the medical records of 36 patients were lost. In total, 926 patients were included in the study. Six hundred sixty-two patients received general anaesthesia, while 264 patients received epidural anaesthesia. After propensity score matching analysis, 264 patients in the general anaesthesia group were matched with 264 patients in the epidural anaesthesia group. The results showed that the differences in base information between the two groups before and after propensity score matching were not statistically significant (Table 1).

**Table 1:** Baseline information of two groups. Values are given as the mean (SD) or number (proportion)

Covariate	Before propensity match score			After propensity match score	
	Group G *n* = 662	Group E *n* = 264	*P* valve	Group G *n* = 264	*P* valve
Age	58.32 ± 12.703	56.62 ± 13.809	0.073	58.54 ± 11.968	0.088
Height	165.45 ± 6.903	165.945 ± 7.090	0.329	165.417 ± 7.102	0.393
Weight	63.66 ± 10.144	63.364 ± 10.608	0.693	63.520 ± 10.706	0.866
Gender			0.076		0.170
male	571 (83.6%)	239 (90.5%)		229 (86.7%)	
female	91 (13.7%)	25 (9.5%)		35 (13.3%)	
Hypertension			0.095		0.662
yes	159 (24.0%)	50 (18.9%)		54 (20.5%)	
no	503 (76.0%)	214 (81.1%)		210 (79.5%)	
Diabetes mellitus			0.495		0.579
yes	43 (6.5%)	14 (5.3%)		17 (6.4%)	
no	619 (93.5%)	250 (94.7%)		247 (93.6%)	
TNM stage			0.697		0.609
TaN0M0	507 (76.6%)	199 (74.5%)		204 (77.3%)	
T1N0M0	155 (23.4%)	65 (24.6%)		60 (22.7%)	
Grade			0.060		0.226
Low malignant potential	155 (23.4%)	74 (28.0%)		63 (23.9%)	
Low-grade papillary urothelial carcinoma	291 (44.0%)	124 (47.0%)		118 (44.7%)	
High-grade papillary urothelial carcinoma	216 (32.6%)	66 (25.0%)		83 (31.4%)	
Tumour size			0.169		0.786
< 3 cm	450 (68.0%)	167 (63.3%)		170 (64.4%)	
≥ 3 cm	212 (32.0%)	97 (36.7%)		74 (35.6%)	
Number of tumour			0.150		0.207
Solitary	432 (65.3%)	159 (60.2%)		173 (65,5%)	
Multiple	230 (34.7%)	105 (39.8%)		91 (34.5%)	
Risk group			0.163		0.500
High-risk tumour	267 (40.3%)	92 (34.8%)		105 (39.8%)	
Intermediate-risk tumour	187 (28.2%)	90 (34.1%)		82 (31.1%)	
Low-risk tumour	208 (31.4%)	82 (31.1%)		77 (29.2%)	
Intravesical chemotherapy			0.976		0.595
yes	264 (39.9%)	105 (39.8%)		111 (42.0%)	
no	398 (60.1%)	159 (60.2%)		153 (58.0%)	

### Relationship between anaesthesia techniques and recurrence before and after the propensity score matching

**Fig. 1:**
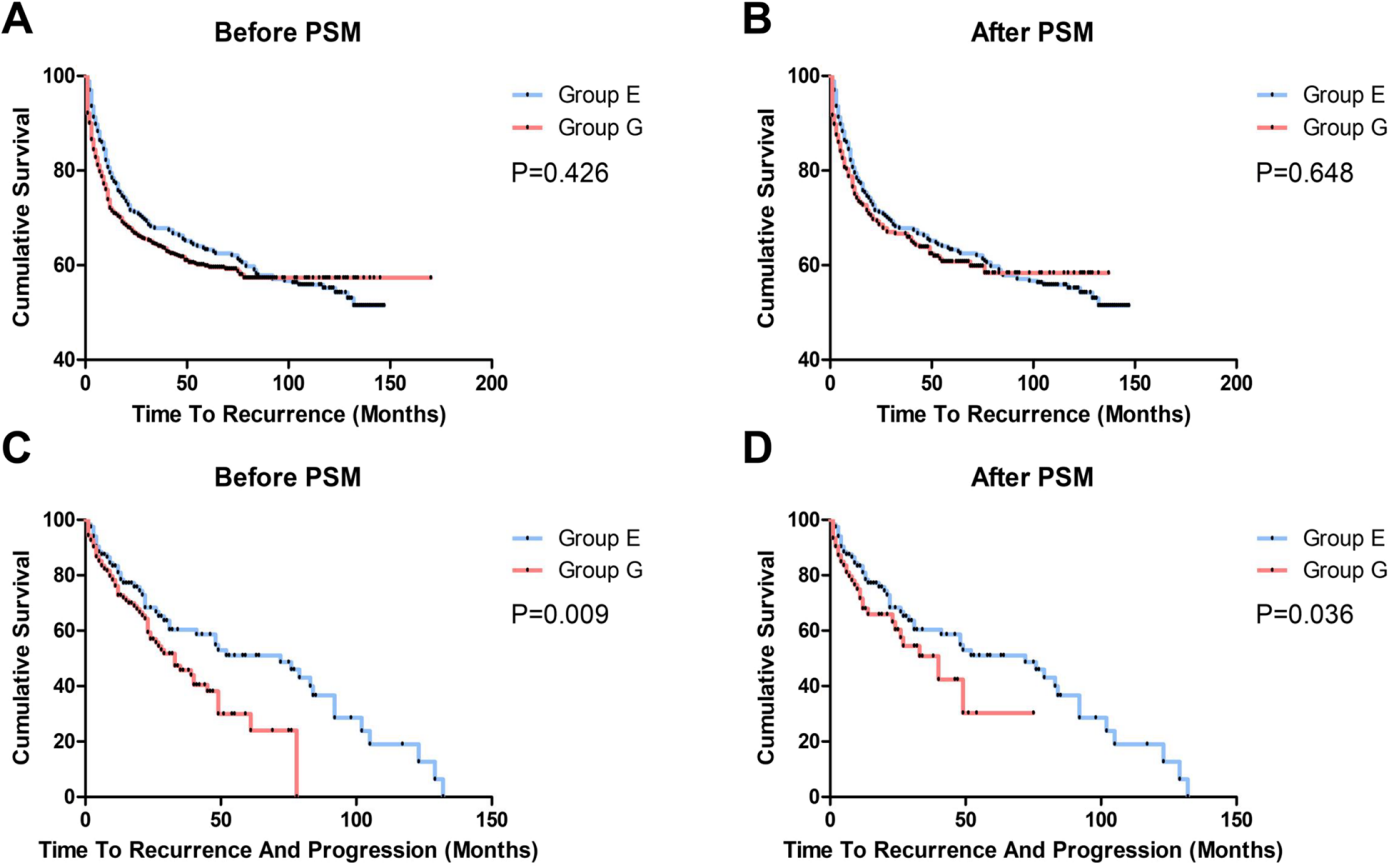
Kaplan-Meier survival curves of two groups. Recurrence-free survival time between group G and group E before (**A**) and after (**B**) PSM was no significant difference. Progression-free survival time before (**C**) and after (**D**) PSM was significant difference between two groups. *P* Value is determined by log-rank test

The total recurrence rate and mean recurrence-free survival times of group G and group E were 40.2%, 104.458 ± 3.170 months and 45.5% (*P* = 0.142), 93.343 ± 3.820 months, respectively. 1-year, 2-year and 3-year recurrence rate of group G were27.9, 33.2 and 36%, as well as that of group E were 20.5, 28.8 and 32.2%. After propensity score matching, the total recurrence rate and mean recurrence-free survival times of group G were 39.0% (*P* = 0.134), 87.329 ± 3.859 months and 1-year, 2-year, 3-year recurrence rate were 25, 31.4, and 33.3%. The results of Kaplan-Meier survival estimates showed that recurrence-free survival time did not differ significantly between the two groups before and after propensity score matching. (Fig. 1, *P* = 0.426, *P* = 0.648).

Univariable Cox regression analysis for recurrence-free survival time showed that age, gender, number of tumour, TNM stage, grade, risk group and intravesical chemotherapy were associated with recurrence, but after propensity score matching, age, number of tumour, TNM stage, grade, risk group and intravesical chemotherapy were associated with recurrence (Table 2). The results of multivariable Cox regression analysis indicated that age, tumour grade, number of tumour and intravesical chemotherapy were significantly associated with recurrence-free survival time before and after the propensity score matching, but the other covariates that were associated with recurrence in the univariable Cox regression analysis were not significantly associated with recurrence-free survival time (Table 3).

**Table 2:** Univariable Cox regression analysis of recurrence

Covariate	Before propensity match score		After propensity match score	
	HR (95%CI)	*P* value	HR (95%CI)	*P* value
Age	1.035 (1.026–1.043)	**< 0.001**	1.028 (1.017–1.039)	**< 0.001**
Gender	0.696 (0.496–0.977)	**0.036**	0.665 (0.416–1.065)	0.089
Hypertension	1.211 (0.963–1.524)	0.101	1.067 (0.773–1.473)	0.692
Diabetes mellitus	1.148 (0.771–1.710)	0.496	0.933 (0.521–1.669)	0.814
Number of tumour	2.243 (1.836–2.741)	**< 0.001**	2.547 (1.954–3.318)	**< 0.001**
TNM stage	1.950 (1.577–2.410)	**< 0.001**	1.515 (1.136–2.021)	**0.005**
Grade	1.690 (1.464–1.952)	**< 0.001**	1.451 (1.205–1.747)	**< 0.001**
Tumour size	1.193 (0.970–1.468)	0.095	1.029 (0.784–1.352)	0.836
Risk group	0.583 (0.513–0.663)	**< 0.001**	0.679 (0.576–0.802)	**< 0.001**
Intravesical chemotherapy	0.741 (0.600–0.915)	**0.005**	0.727 (0.552–0.959)	**0.024**
Anaesthesia techniques	1.093 (0.876–1.363)	0.431	1.064 (0.813–1.393)	0.650

**Table 3:** Multivariable Cox regression analysis of recurrence

Covariate	Before propensity match score		After propensity match score	
	HR (95%CI)	*P* value	HR (95%CI)	*P* value
Age	1.024 (1.015–1.034)	**< 0.001**	1.018 (1.007–1.030)	**0.002**
Gender	0.729 (0.519–1.024)	0.069	_	
TNM stage	1.287 (0.981–1.687)	0.068	1.141 (0.782–1.663)	0.495
Grade	1.405 (1.173–1.684)	**< 0.001**	1.360 (1.065–1.735)	**0.014**
Number of tumour	1.928 (1.554–2.390)	**< 0.001**	2.387 (1.788–3.188)	**< 0.001**
Risk group	0.911 (0.737–1.128)	0.394	1.026 (0.767–1.373)	0.864
Intravesical chemotherapy	0.714 (0.576–0.885)	**0.002**	0.688 (0.519–0.912)	**0.009**

### Relationship between anaesthesia techniques and progression before and after the propensity score matching

Three hundred eighty-six patients suffered recurrence after TURBT surgery, 266 of them accepted general anaesthesia. The mean progression-free survival time of group G was 37.775 ± 2.931 months, and that of group E was 63.277 ± 5.701 months, the progression rate of group G was 31.2%, that of group E was 42.5% (*P* = 0.031). After the propensity score matching, 120 patients in general anaesthesia group were matched with 120 patients in epidural anaesthesia group, and the mean progression-free survival time of group G was 38.214 ± 4.189 months, progression rate of group G was 30.8% (*P* = 0.134). The Kaplan-Meier survival curves for progression-free survival showed that the difference between the two groups was statistically significant before and after the propensity score matching (Fig. 1, *P* = 0.009, *P* = 0.036).

In the univariate analysis, age, TNM stage, grade, tumour size, risk group, and anaesthesia techniques were associated with progression. After the propensity score matching, age, diabetes mellitus, TNM stage, grade, tumour size, risk group and anaesthesia techniques were associated with progression (Table 4). The results of multivariable Cox regression analysis showed that age, grade, and tumour size were significantly associated with progression. However, after the propensity score matching, only grade remained significant (Table 5).

**Table 4:** Univariable Cox regression analysis of progression

Covariate	Before propensity match score		After propensity match score	
	HR (95%CI)	*P* value	HR (95%CI)	*P* value
Age	1.019 (1.005–1.034)	**0.010**	1.021 (1.003–1.039)	**0.020**
Gender	0.601 (0.337–1.073)	0.085	0.543 (0.259–1.139)	0.106
Hypertension	1.051 (0.715–1.544)	0.801	1.206 (0.750–1.939)	0.440
Diabetes mellitus	1.370 (0.716–2.622)	0.341	2.617 (1.248–5.488)	**0.011**
Number of tumour	0.868 (0.617–1.220)	0.415	1.013 (0.661–1.551)	0.954
TNM stage	2.047 (1.439–2.911)	**< 0.001**	2.323 (1.502–3.593)	**< 0.001**
Grade	1.836 (1.441–2.339)	**< 0.001**	1.952 (1.451–2.625)	**< 0.001**
Tumour size	1.732 (1.228–2.443)	**0.002**	1.583 (1.029–2.438)	**0.037**
Risk group	0.633 (0.494–0.811)	**< 0.001**	0.560 (0.411–0.764)	**< 0.001**
Intravesical chemotherapy	0.826 (0.573–1.190)	0.305	0.712 (0.453–1.119)	0.141
Anaesthesia techniques	1.664 (1.128–2.455)	**0.010**	1.616 (1.022–2.555)	**0.040**

**Table 5:** Multivariable Cox regression analysis of progression

Covariate	Before propensity match score		After propensity match score	
	HR (95%CI)	*P* value	HR (95%CI)	*P* value
Age	1.016 (1.001–1.031)	**0.034**	1.016 (0.998–1.034)	0.087
Diabetes mellitus	–		2.067 (0.967–4.421)	0.061
TNM stage	1.484 (0.948–2.321)	0.084	1.369 (0.740–2.531)	0.317
Grade	1.772 (1.235–2.541)	**0.002**	1.743 (1.126–2.699)	**0.013**
Tumour size	1.441 (1.006–2.065)	**0.046**	1.279 (0.811–2.018)	0.290
Risk group	1.254 (0.855–1.840)	0.247	1.086 (0.660–1.787)	0.745
Anaesthesia techniques	1.453 (0.976–2.163)	0.066	1.523 (0.953–2.433)	0.079

## Discussion

The status of the immune system, particularly cellular immunity, can influence the ability of malignant cells to proliferate. Thus, if the immune system of a patient is suppressed, residual malignant cells are more likely to proliferate and spread. Previous study indicated that the anaesthetics and adjuvants used in the perioperative period could affect the balance of the immune system [15]. A number of retrospective studies have demonstrated that surgery and inflammation can influence the neuroendocrine response, which may negatively affect the function of the T cells [16, 17]. Additionally, anaesthesia techniques can inhibit cellular immunity by interfering with nervous and endocrine system [18–20].

The CD3+, CD4+ and CD8+ T cells play critical roles in antitumour immunity [21]. The benefit of epidural anaesthesia on T-cell immunity was reported in a study of gastric cancer resection. In this clinical trial, CD3+ T cells decreased more in the general anaesthesia group than in the combination of general and epidural anaesthesia group [22]. The study on general anaesthesia indicated that the tumour cytotoxicity of natural killer (NK) cells, which play a crucial role in anti-tumour immunity, was inhibited by volatile anaesthetics including isoflurane and sevoflurane [23]. After laparoscopic radical hysterectomy surgery for cervical cancer, the counts of circulating lymphocytes (CD3+ cells, CD4+ cells, and NK cells), as well as the CD4+ to CD8+ cell ratio were significantly lower in the sevoflurane group than in the propofol group, and compared with baseline, the numbers of circulating lymphocytes other than CD8+ cells decreased significantly in both groups [24]. CD4+ cells, CD8+ cells and the CD4+/CD8+ ratio have significant functional implications for cell-mediated immunity. In particular, the CD4+/CD8+ ratio is considered to be positively associated with the function of cell-mediated immunity [25].

However, a study regarding the effects of different anaesthetic methods on the immune function of patients undergoing primary liver cancer resection found that postsurgical levels of CD4+ T cells and CD8+ T cells were lower after general anaesthesia combined with epidural anaesthesia than after general anaesthesia alone [26]. The CD4+ and CD8+ T cells have important anti-tumour immune functions [21], thus combined anaesthesia had a negative influence on cellular immunity. The results of many clinical trials have also shown no significant benefit of epidural anaesthesia compared to general anaesthesia in terms of survival prognosis. A study in patients with gallbladder cancer suggested that although the general and epidural combined anaesthesia could improve cellular immunity after laparoscopic cholecystectomy, the 1-year, 2-year and 3-year survival rates did not significantly differ compared with general anaesthesia [27]. A multicentre, prospective, randomized study shown that epidural combined anaesthesia had no effect on survival of colon cancer patients with distant metastases [9]. No difference was observed between the epidural and general groups in disease-free survival after radical prostatectomy [28]. Many clinical randomized trials on breast cancer and regional or general anaesthesia confirmed the result that the reginal anaesthetic including epidural techniques did not affect the immunological process associated with recurrence, metastasis or mortality, indicating that regional anaesthesia was not superior to general anaesthesia in the survival condition after breast cancer surgery [13, 29–31]. In 2011, a large multicenter randomized clinical trial on epidural anesthesia and recurrence-free survival time after abdominal tumor surgery was completed by 23 hospitals in Australia, New Zealand, and Asia. The study, which followed 503 patients over time after surgery, showed that use of epidural block in abdominal surgery for cancer was not associated with improved cancer-free survival [32]. The conclusion that epidural anesthesia had no effect on the prognosis of the tumor was also verified by our research.

In our study, we investigated whether general anaesthesia (group G) versus epidural anaesthesia (group E) could influence the prognosis of TURBT in patients with primary non-muscle-invasive bladder cancer. The final results revealed that neither the recurrence rate nor the recurrence-free survival time differed significantly between the two groups, and anaesthesia techniques had no effect on recurrence. Furthermore, among relapsed patients, there was no difference in progression rates between the two groups, but progression-free survival time of group E was longer. However, multivariable Cox regression analysis after propensity score matching indicated that, among the factors examined, only grade was associated with the progression-free survival time. Consequently, this study demonstrated that general anaesthesia and epidural anaesthesia did not affect the recurrence and progression of patients with non-muscle-invasive bladder cancer after TURBT.

As previously reported, propofol, isoflurane, and sevoflurane, which are commonly used in general anaesthesia, negatively affect the immune function of patients undergoing tumour surgery. It seemed that epidural anaesthesia without general anaesthesia drugs might improve outcomes for patients with tumours. However, in this study, the two anaesthesia techniques resulted in similar outcomes. The biological behaviour of cancer cells and the prognosis of patients are impacted by many factors. Although epidural anaesthesia might be better than general anaesthesia in terms of changing immune function, the primary factors determining bladder cancer prognosis are the biological characteristics of the cancer cells and the use of other treatment methods, such as intravesical chemotherapy.

## Conclusion

In this retrospective study, patients in the epidural anaesthesia group did not have a lower recurrence and progression rate. Progression-free survival time of group E was longer. Anaesthesia technique was not an independent risk factor for prognosis. General anaesthesia provides a more comfortable experience for patients and can reduce anxiety and fear during surgery. For the surgeons, general anaesthesia provides a more appropriate depth of sedation and analgesia as well as more satisfactory muscle relaxation, alleviating concerns and distractions during surgery. Results of this study can offer some guidance to anaesthesiologists and reduce interference in selecting of anaesthesia techniques.

## Data Availability

The datasets generated and/or analyzed during the current study are not publicly available due hospital regulation, but are available from the corresponding author on reasonable request.

## Abbreviations

TURBT: Transurethral bladder tumour resection
CIS: Carcinoma in situ
TNM: Tumour, node, metastases
NK: Natural killer
PSM: Propensity score match
CD: Cluster of differentiation
Group G: General anaesthesia group
Group E: Epidural anaesthesia group
RFS: Recurrence-free survival
PFS: Progression-free survival
BCG: Bacillus Calmette-Guerin
